# Assessing Orofacial Esthetics in Prosthodontic Care: A Systematic Review and Meta-Analysis of Psychometric Properties

**DOI:** 10.12669/pjms.40.8.9860

**Published:** 2024-09

**Authors:** Zara Amir, Jacob John, Mahmoud Danaee, Zamros Yuzadi Mohd Yusof

**Affiliations:** 1 Zara Amir, MDSc Student, Department of Community Oral Health, Clinical Prevention, Faculty of Dentistry, Universiti Malaya, Kuala Lumpur, Malaysia; 2 Jacob John, Department of Restorative Dentistry, Community Oral Health Research Group, Faculty of Dentistry, Universiti Malaya, Kuala Lumpur, Malaysia; 3 Mahmoud Danaee, Department of Social and Preventive Medicine, Faculty of Medicine, Universiti Malaya, Kuala Lumpur, Malaysia; 4 Zamros Yuzadi Mohd Yusof, Department of Community Oral Health and Clinical Prevention, Faculty of Dentistry, Community Oral Health Research Group, Faculty of Dentistry, Universiti Malaya, Kuala Lumpur, Malaysia

**Keywords:** Orofacial esthetic, Orofacial aesthetic, Psychometric

## Abstract

**Objective:**

To validate and ascertain the reliability of the Orofacial Esthetic Scale specifically within the context of prosthodontic patients, aiming to establish it as a credible and standardized tool for evaluating orofacial esthetics in this specialized cohort.

**Methods:**

The systematic analysis adhered to the guidelines outlined by the Preferred Reporting Items for Systematic Reviews and Meta-Analyses (PRISMA). A systematic search using four databases PubMed /Medline, Web of Science, Scopus, Science Direct along with manual and grey literature (ProQuest and Google scholar) till 30 October 2023 was performed. The risk of biasness was assessed using Joanna Briggs Institute (JBI) critical appraisal checklist followed by validity analysis. For meta-analysis, psychometric values (Cronbach alpha and Intra class correlation coefficient) were used through MedCalc software.

**Results:**

The overall ICC value was 0.95 (95% CI: 0.94 to 0.96) and the overall Cronbach alpha value was 0.94 (95% CI: 0.92 to 0.96). There was evidence of high heterogeneity and no publication bias among the studies included.

**Conclusion:**

This study underscores the pivotal role of orofacial esthetics in prosthodontic care, introducing the Orofacial Esthetic Scale as a validated tool to quantitatively assess subjective perceptions. This will highlight its potential for tailored treatment planning and improved patient outcomes.

## INTRODUCTION

Prosthodontic patients constitute a unique cohort within dental care necessitate tailored treatment plans to address complex dental and facial concerns effectively.^1^ Understanding the specific needs and intricacies of prosthodontic patients is fundamental in delivering effective and personalized care within this specialized domain of dentistry. The significance of esthetics in prosthodontic care is closely linked to an individual’s self-confidence, psychological outlook, and, ultimately, their overall quality of life.^2^ As a result, assessing `esthetics during oral reconstruction should not solely rely on individual preferences, whether from the clinician or patient.

The patient’s subjective assessment of their oral health, including their personal perception of esthetics, holds considerable importance in evaluating treatment effectiveness.^3^ This subjective aspect poses a challenge in assessing esthetics because the ultimate outcome needs to align with the patient’s specific esthetic preferences, which should be one of the treatment outcomes measured in prosthodontic care. To streamline and enhance this process, there is a critical need for valid and reliable tools to standardize and expedite the evaluation of esthetics. Therefore, Mursid et al., conducted a scoping review to assess various instruments used for esthetics and concluded that OES is the most widely used instrument for self-evaluation in orofacial esthetics research.^4^

The Orofacial Esthetic Scale (OES) is a validated tool used to assess and measure the esthetic perceptions of individuals regarding various aspects of the oral and facial region. The 8-item instrument have seven questions directly asking on esthetics pertaining to face, facial profile, mouth, rows of teeth, tooth shape/form, tooth color, gums and one global assessment question about the overall perception towards the face, mouth, and teeth.^5^ It measures the individual’s orofacial esthetics perception using an 11-point Likert scale (0-70). The first seven items are combined into a summary score ranging from 0 (worst score, patients are very dissatisfied with all esthetic items) to 70 (best score, patients are very satisfied with all esthetic items).^6^

The questionnaire was developed based on applying broad esthetic principles to the stomatognathic system. OES, which was originally developed for prosthodontic patients^5^ was further tested on general population by the same author.^7^ Numerous authors have translated OES into several languages, and some have used it as an intervention tool. The absence of validated orofacial esthetics assessment tools in prosthodontic care impedes communication and personalized treatment planning. Thus, this study aimed to evaluate the psychometric properties of the Orofacial Esthetic Scale (OES) within prosthodontic patients, ensuring its reliability and applicability for quantifying subjective esthetic perceptions in this clinical context.

## METHODS

The methodology was based on the guidance by PRISMA and Cochrane Handbook for Systematic Review.^8^ The focus question was formulated to determine the overall assessment score in prosthodontic patients based on “PICO” as follow:

“Is Orofacial Esthetic Scale a valid and reliable instrument to use for prosthodontic patients?”

P = prosthodontic patients

I= OES questionnaire

C = psychometric scores of OES

O = measure validity and reliability of OES

The authors extracted articles from the PubMed/Medline, Scopus, Web of Science, Science Direct databases using the meSH terms and filtered them from 2010 to 2023. The detailed search strategy for PubMed/Medline database in advanced search was as follows:

1 = “Orofacial esthetic” OR “Orofacial aesthetic”,

2 = “Scale” OR “Questionnaire” OR “Survey” OR “Development” OR “Validation” OR “Psychometric”; and then combining #1 AND #2, using the filters “2010- 2023” and “English”. Each database was last searched on 30 October 2023.

After initial identification of 2044 related publications through databases, duplicates were removed using EndNote (version X9; Thomson Reuters, New York, NY). References were then manually cross-reviewed for each title and abstract to identify studies fulfilling the set inclusion/exclusion criterion by two authors. In addition to database searches, a thorough grey literature search was conducted. Authors also scanned reference lists in articles and reviews, and related database entries.

### Inclusion criteria:

- Studies that have used OES questionnaire in prosthodontic patients.
- Studies that assessed reliability and validity of OES in prosthodontic patients.
- All study designs were included.
- Studies published since 2010.
- Studies written in English.
- Studies that have been published.

### Exclusion criteria:

- Studies that compared the validity of OES with a distinct tool that measured orofacial esthetics.
- Review articles, proposal papers and articles whose full texts were not available.

Reliability and validity values were digitized. Internal consistency was assessed using Cronbach’s alpha, while test-retest reliability employed interclass correlation (ICC). Convergent validity was determined through correlation coefficients with OES comparator measures, and construct validity was analyzed via factor analysis, KMO values, and the number of factors.

### Assessment of the quality and bias risk

Two authors independently assessed bias using the Joanna Briggs Institute (JBI) critical appraisal checklists. Specifically, the JBI checklist for case control studies was employed. Items were rated as “yes,” “no,” “unclear,” or “inapplicable.” Studies scoring over 70% “yes” were deemed low bias risk, 50-69% moderate, and below 49% high.^9^

### Statistical analysis of the included studies

Meta-analysis was conducted on Cronbach’s alpha and ICC reliability scores. Convergent validity couldn’t undergo meta-analysis due to differing survey items. Construct validity was qualitatively assessed due to lack of recognized methodology. Content and discriminative analysis were performed using MedCalc-version 19.5.3 which calculates the weighted summary correlation coefficient under the random effects model using the Hedges-Olkin method and a Fisher Z transformation of the correlation coefficients. Cochrane guidelines interpret heterogeneity as follows: 0-40% not significant, 30-60% moderate, 50-90% substantial, 75-100% considerable.^10^

## RESULTS

**Fig.1:**
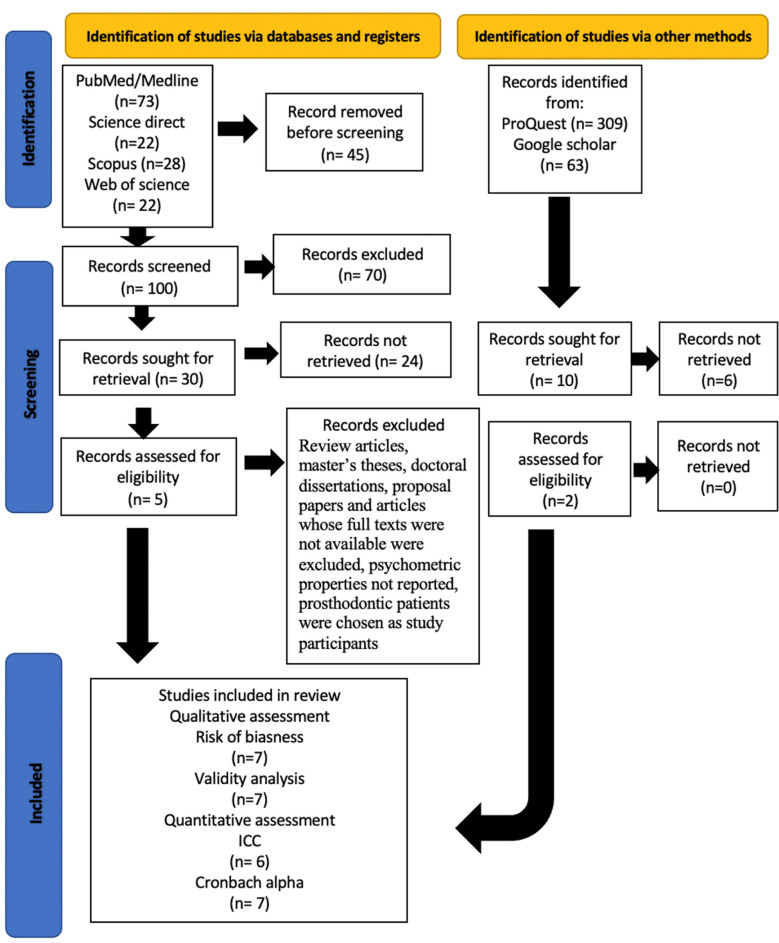
Flowchart of the selection of studies for the review.

A flowchart describing the systematic review search results is presented in Fig.1. Of the 2044 articles, seven peer-reviewed ones were selected, all with a case-control design. They involved 1144 prosthodontic patients. For the psychometric evaluation, seven articles tested for Cronbach alpha, six for test-retest, five for construct validity, seven for convergent validity, six for discriminant validity and two for content validity.

For convergent validity five studies compared three items of OHIP questionnaire with r = 0.43 to 0.72,^6^ 0.73 to 0.81,^11^ 0.83 to 0.70,^12^ 0.65 to 0.88^13^ and 0.67 to 0.81,^14^ respectively and reported significant p value. The study by Reissmann et al.^15^ calculated the correlation between the instrument’s summary score with the patients’ global assessment of their esthetics (r = 0.87) and external ratings of an expert group (r = 0.55). The study by Edoardo et al.^16^ calculated the correlation between OES scores of prosthodontic patients and clinicians with r = 0.93. Construct validity was calculated by factor analysis; one study^12^ used both confirmatory factor analysis (CFA) and exploratory factor analysis (EFA), with the factor loading from 0.892 to 0.933.

Three studies used EFA only, with factor loading of 0.45 to 0.80,^15^ 0.74 to 0.94^14^ respectively and the values for one article^13^ were not given, while one study^16^ used CFA only. All the studies revealed unidimensional structure. Discriminant validity was calculated in four studies^11^-^14^ using one-way analysis of variance (ANOVA) and Sheffé post hoc test while two studies^6^,^15^ calculated by two sample t-test. Only two studies calculated content validity: one study^6^ with 119 respondents and the other^12^ with a sample size of four experts.Table-I summarized the types of validity calculated in each article.

**Table-I:** Validity parameters of Orofacial Esthetic Scale tool administered to identify patient dental esthetics needs.

Study ID	Content validity	Concurrent validity	Construct validity	Convergent validity	Discriminative validity
Larsson, P (2010)^10^	119	NA	NA	Spearman rank correlation	t-test
Persic, S. (2011)^18^	NA	NA	NA	Spearman rank correlation	one-way ANOVA & Sheffé post hoc test
Zhao, Y. (2013)^19^	4 experts	NA	CFA, EFA KMO=0.939, one factor model	Pearson correlation coefficient	one-way ANOVA & Sheffe post hoc test
Bimbashi, V. (2015^)20^	NA	NA	EFA, KMO=0.921, one factor model	Spearman rank correlation.	One way ANOVA
Reissmann, DR. (2015)^21^	NA	NA	EFA one factor model	Pearson correlation coefficient	two sample t tests Cohen’s d, a standardized effect size
Alhajj, M.N. (2017)^22^	NA	NA	EFA One factor model, KMO=0.87	Spearman’s rank correlations	one-way ANOVA & Sheffe post hoc test
Edoardo, R. (2023)^24^	NA	NA	CFA	Pearson correlation coefficient	NA

NA – not available.

The JBI assessment checklist (Table-II) shows that three studies^6^,^11^,^12^ were ranked as low risk of bias, three^14^-^16^ were moderate and only one study^13^ was high. The domains with the greatest number of methodological flaws were related to the control of confounding factors and strategies to deal with them. However, almost all the studies used appropriate statistical analysis according to their study requirements.

**Table-II:** Risk of bias using Joanna Briggs Institute Critical Assessment Checklist for Case control studies.

STUDY ID	Q1	Q2	Q3	Q4	Q5	Q6	Q7	Q8	Q9	Q10	%
Larsson, P (2010)^10^											70.0%
Persic, S. (2011)^18^											70.0%
Zhao, Y. (2013)^19^											70.0%
Bimbashi, V. (2015)^20^											40.0%
Reissmann, D. R. (2015)^21^											60.0%
Alhajj, M.N. (2017)^22^											60.0%
Edoardo, R. (2023)^23^											60.0%

Q1. Were the groups comparable other than the presence of disease in cases or the absence of disease in controls? Q2. Were cases and controls matched appropriately? Q3. Were the same criteria used for identification of cases and controls? Q4. Was exposure measured in a standard, valid and reliable way? Q5. Was exposure measured in the same way for cases and controls? Q6. Were confounding factors identified? Q7. Were strategies to deal with confounding factors stated? Q8. Were outcomes assessed in a standard, valid and reliable way for cases and controls? Q9. Was the exposure period of interest long enough to be meaningful? Q10. Was appropriate statistical analysis used?

Among the seven studies that calculated psychometric properties of the OES on the prosthodontic patients, only six (of which one study calculated two values of ICC) with the same study design provided information on the ICC scores with overall ICC of 0.95 (95% CI: 0.94 to 0.96) and seven studies reported Cronbach alpha values with overall value of 0.94 (95% CI: 0.92 to 0.96, indicating excellent internal consistency. Heterogeneity was significant in both analyses (p = <.0001), with OES score variations of 76.32% for ICC group and 88.41% for Cronbach alpha group respectively. Bias due to small study effect was tested using Begg’s test and Egger’s test. The two tests showed no evidence of bias (P = 0.60 and 0.36 for the ICC group and 0.51 and 0.51 for the Cronbach alpha group). Funnel plots showed no publication bias in both meta-analyses (Fig.3).

**Fig.2:**
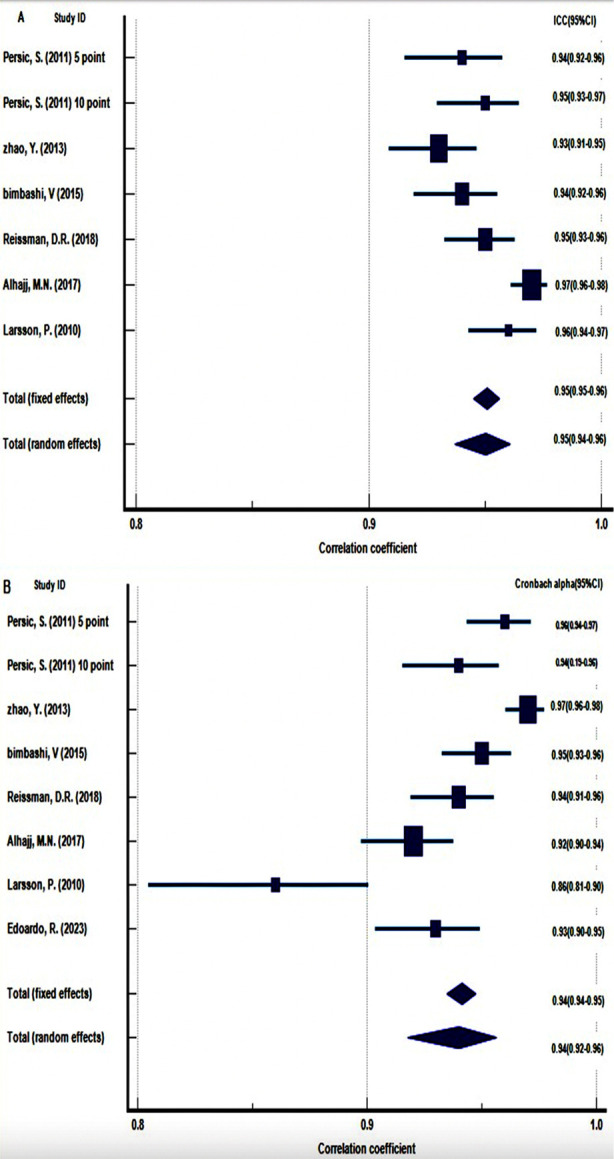
Forest plot depicting the results of meta-analysis for, (A) studies including ICC values. (B) studies including Cronbach alpha values.

**Fig.3:**
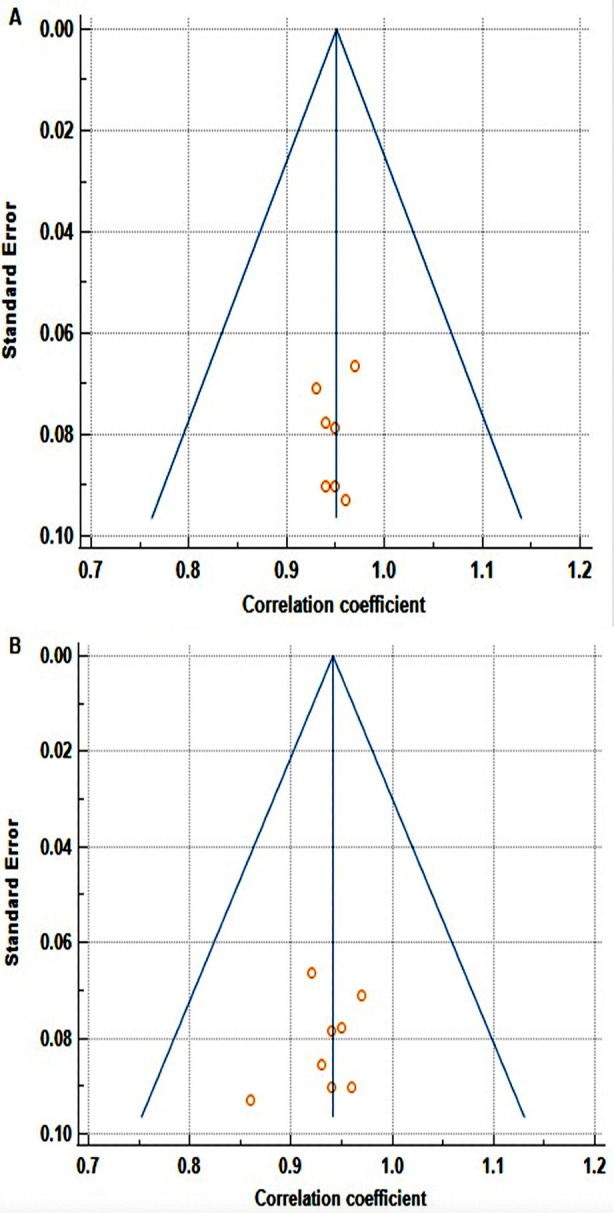
Funnel plot for evaluation of publication bias for, (A) studies with ICC values. (B) Studies with Cronbach alpha values.

## DISCUSSION

This systematic review and meta-analysis thoroughly evaluated the OES among prosthodontic patients. Seven articles, all with a case-control design, were included, comprising 1144 patients. Psychometric evaluation covered Cronbach’s alpha, test-retest reliability, construct validity, convergent validity, discriminant validity, and content validity.

This study revealed that four studies were rated as low risk of bias, indicating high-quality, while three were moderate, and only one recorded with the lowest score of 40%. Overall, evidence supports OES validity and reliability in assessing orofacial esthetics among prosthodontic patients. This review qualitatively analyzed OES validity methods across articles, confirming construct validity through structural, convergent, and known-groups validity. All items were good indicators for the measured construct, correlated well with other measures of the same construct, and differentiated well between subjects with known differences.

Additionally, these publications supported OES unidimensionality, adhering to traditional methods of sample size involving 5-20 respondents per item for the factor analysis.^17^ The pooled ICC and Cronbach fell within the recommended ranges 0.70 – 0.95 values,^18^ signify substantial agreement and coherence within the scale items, validating its application in clinical and research settings. The meta-analyses indicated significant heterogeneity among studies, reflecting variations across population and countries. This underscores the need for cross-cultural adaptation. Moreover, absence of publication bias enhances credibility of findings within the published literature.

### Limitation

The limitation of this review includes reliance on a restricted number of studies for meta-analysis, necessitating updates with more data in the future. Additionally, English-only search criteria may introduce language bias, potentially omitting other relevant evidence. The strength of this review lies in it being the first meta-analysis to explore orofacial esthetics perception globally among prosthodontic patients using the OES questionnaire. Dentists can utilize this tool for better treatment planning and understanding patient needs and perceptions.

## CONCLUSION

This comprehensive analysis consolidates the evidence supporting the OES as a valid and reliable instrument for assessing orofacial esthetics in prosthodontic patients. The robust psychometric properties, highlighted by the high ICC and Cronbach’s alpha values, signify its potential utility in both research and clinical settings. Future studies might benefit from exploring the sources of heterogeneity observed and validating the scale in diverse populations to enhance its applicability and generalizability. The findings also provide valuable insights into the assessment of orofacial esthetics and lay a strong foundation for employing the OES as a standardized tool in prosthodontic care, facilitating better treatment planning and patient-centered care.

### Authors’ contribution:

**ZA:** Conceived and designed the study, collected the data and performed data analysis, prepared the manuscript.

**JJ, MD & ZY:** Conceived and designed the study, helped in analyzing the data, reviewed and provided final approval.

All authors are responsible for the accuracy and integrity of the manuscript.
